# Determinants of growth after kidney transplantation in prepubertal children

**DOI:** 10.1007/s00467-021-04922-2

**Published:** 2021-02-23

**Authors:** Julia Grohs, Rainer-Maria Rebling, Kerstin Froede, Kristin Hmeidi, Leo Pavičić, Jutta Gellermann, Dominik Müller, Uwe Querfeld, Dieter Haffner, Miroslav Živičnjak

**Affiliations:** 1 Department of Pediatric Kidney, Liver and Metabolic Diseases, Children’s Hospital, Hannover Medical School, Carl-Neuberg-Str. 1, 30625 Hannover, Germany; 2 Department of Orthopedic Surgery, Vivantes Auguste-Viktoria-Hospital, Rubensstr. 125, 12157 Berlin, Germany; 3 Rendiceva 28B, 10000 Zagreb, Croatia; 4 Department Department of Pediatrics, Division of Gastroenterology, Nephrology and Metabolic Diseases, Charité - Universitätsmedizin Berlin, Campus Virchow-Klinikum, Augstenburger Platz 1, 13353 Berlin, Germany

**Keywords:** Chronic kidney disease, Children, Kidney transplantation, Growth, Body proportions, CKD-MBD

## Abstract

**Background:**

Short stature is a frequent complication after pediatric kidney transplantation (KT). Whether the type of transplantation and prior treatment with recombinant human growth hormone (GH) affects post-transplant growth, is unclear.

**Methods:**

Body height, leg length, sitting height, and sitting height index (as a measure of body proportions) were prospectively investigated in 148 prepubertal patients enrolled in the CKD Growth and Development study with a median follow-up of 5.0 years. We used linear mixed-effects models to identify predictors for body dimensions.

**Results:**

Pre-transplant *Z* scores for height (− 2.18), sitting height (− 1.37), and leg length (− 2.30) were reduced, and sitting height index (1.59) was increased compared to healthy children, indicating disproportionate short stature. Catch-up growth in children aged less than 4 years was mainly due to stimulated trunk length, and in older children to improved leg length, resulting in normalization of body height and proportions before puberty in the majority of patients. Use of GH in the pre-transplant period, congenital CKD, birth parameters, parental height, time after KT, steroid exposure, and transplant function were significantly associated with growth outcome. Although, unadjusted growth data suggested superior post-transplant growth after (pre-emptive) living donor KT, this was no longer true after adjusting for the abovementioned confounders.

**Conclusions:**

Catch-up growth after KT is mainly due to stimulated trunk growth in young children (< 4 years) and improved leg growth in older children. Beside transplant function, steroid exposure and use of GH in the pre-transplant period are the main potentially modifiable factors associated with better growth outcome.

## Introduction

Growth failure is frequent in children with stage 5 chronic kidney disease (CKD 5). Its etiology is multifactorial and includes intrauterine growth restriction, malnutrition, mineral and bone disorder, acidosis, fluid and electrolyte abnormalities, and disturbances of the somatotropic hormone axis [1]. Registry data shows that growth outcome has improved considerably in these patients during recent decades [2, 3]. However, reduced adult height is still noted in approximately 40% of children with CKD 5; it not only affects quality of life, self-esteem, and social rehabilitation, but is also associated with excessive mortality in these patients [3–8].

Kidney transplantation (KT) resolves many of the metabolic and endocrine disorders contributing to uremic growth failure, but rarely results in substantial catch-up growth [5, 9, 10]. Young age at KT, transplant function and steroid exposure are important factors influencing post-transplant growth, but they only partly explain the large variability of growth outcome [5, 9, 11–14]. Whether the type of transplantation (living versus deceased donor; pre-emptive KT versus prior dialysis) or treatment with recombinant human growth hormone (GH) prior to KT impact on post-transplant growth are largely unknown [5, 10, 15]. Previous analyses mainly coming from patient registries did not include detailed data on factors affecting growth, such as abnormal birth history, low parental height, pubertal status, and medication use (i.e., steroids, GH) and could therefore not adjust for these variables [13, 16–19]. Finally, CKD 5 in children is associated with more severe impairment of leg growth in comparison to trunk growth resulting in disproportionate short stature [13, 19, 20]. We hypothesized that KT stimulates the growth of linear body segments (trunk and legs) differentially in toddlers and older children and that its effects on body proportions are therefore age dependent.

To this aim, we assessed post-transplant changes in linear body dimensions (height, sitting height, and leg length), and the ratio of trunk length to total body height (sitting height index) as a measure of body proportion and its determinants in 148 prepubertal children enrolled in the prospective CKD Growth and Development Study.

## Methods

### Study design and population

From May 1998 until November 2019, a total of 947 children with CKD stages 3–5D and after KT were enrolled in the CKD Growth and Development Study, which is a prospective, observational cohort study performed at two pediatric nephrology centers in Germany (Hannover Medical School and Charité Universitätsmedizin Berlin). Patients are followed up at yearly intervals for clinical, biochemical and anthropometric assessment as previously described [19]. The study was approved by the local ethics committees, and research was performed in accordance with the declaration of Helsinki. Study participants and/or their parents gave their consent prior to participation.

For the present analysis, all first-time graft recipients, transplanted before the age of 8 years with a functional kidney transplant and with at least one valid follow-up visit were included. Patients with height-affecting skeletal abnormalities and/or severe locomotor dysfunction (*n* = 16) were excluded. All follow-up visits were included unless the patient reached age of 12 years to avoid bias of puberty. Thus, data from 148 patients (66% male), who underwent 722 annual measurements, with a median follow-up of 5.0 years (IQR, 3–7 years) could be included in the analysis (Table 1).

**Table 1:** Clinical characteristics of 148 prepubertal pediatric kidney transplant recipients

Nonrepeated measurements^a^	% or median	IQR	Range	No. of patients
Male, %	66.2			98 of 148
Age at KT, years	3.68	2.07–5.60	0.49–7.98	148
Age at dialysis initiation, years	1.96	0.59–4.16	0.01–7.81	98 of 148
Age at CKD 5, years	2.77	0.93–4.87	0.01–7.87	148
Duration of dialysis, years	1.02	0.43–1.85	0.01–4.75	98
Pre-emptive KT, %	35.1			52 of 148
Living donor, %	29.3			43 of 147
Congenital CKD, %	79.1			117 of 148
SGA history, %	27.4			37 of 135
GH therapy before KT, %	33.8			50 of 148
Age at start of GH therapy, years	1.93	1.22–4.03	0.27–6.31	50
Duration of GH treatment, years	1.40	0.57–2.23	0.11–5.39	50
Genetic target height, SDS	− 0.13	− 0.79–0.55	− 2.93–1.46	142
Repeated measurements^b^	Estimated marginal mean	95% CI	Range	No. of measurements
eGFR, mL/min per 1.73 m^2^	59	55–63	5–191	666
Steroid dosage, mg/kg per day	0.098	0.086–0.109	0.00–0.67	710
Plasma HCO_3_, mmol/L	22.6	22.0–23.2	18.3–33.3	693
Hemoglobin, g/dL	11.2	11.0–11.4	6.5–15.3	706

*IQR*, interquartile range; *KT*, kidney transplantation; *CKD 5*, stage 5 chronic kidney disease; *SGA*, small for gestational age; *GH*, growth hormone; *SDS*, SD score; *eGFR*, estimated glomerular filtration rate

^a^Basic data (nonrepeated measurements) are given as median and interquartile range (25–75th percentile)

^b^Average values (estimated marginal means) during the observation period are based on all annual values, repeated measurements within the same individual (evaluated with the linear mixed model, random patients and age cohorts)

Children’s personal health care records were used to obtain data on gestational age, umbilical cord artery pH, birth weight, and length. Attainment of CKD 5 was defined by initiation of dialysis or pre-emptive KT. The prescribed dietary intake was in accordance with targeted requirements and estimated glomerular filtration rate (eGFR) was assessed by the Schwartz formula [21]. Newborns were classified as small for gestational age (SGA) if birth weight or length was < 10th percentile according to national growth standards [22]. Primary immunosuppressive protocols included daily prednisolone treatment. Prednisolone dosage was generally tapered down by week 8 to 4 mg/m^2^/day. From 2007, in cases of stable graft function and lack of rejection, patients were regularly weaned off steroids between 6 and 12 months post KT. Overall, steroids were withdrawn in 18% of patients after a median period of 8 months (range 6–12 months). Indicators for the use of GH treatment in the pre- and post-transplant period were a height SD score (SDS) < − 2.0 and a height velocity < 25th percentile. Genetic target height was calculated from mid-parental height [23].

### Anthropometry and outcome variables

Anthropometric measurements were performed by the same investigator (MŽ) as recommended by the International Biologic Program [24] with standardized equipment (Dr. Keller I Stadiometer, Limbach-Oberfrohna, Germany; Siber Hegner Anthropometer, Zürich, Switzerland) as previously described [13]. The sitting height index was calculated as the ratio between sitting height and total body height [25]. SD scores (SDS) for each segment of linear growth were calculated using reference values from healthy children [26, 27].

### Statistical analyses

Data is given as mean ± SD and/or 95% CIs unless otherwise indicated. All anthropometric data is presented as age- and sex-related SDS values. Distribution normality was evaluated by the Shapiro-Wilk test for each parameter. Previous studies demonstrated that both chronological age as well as duration and timing of growth affecting measures, like KT, impact on body growth in children with CKD 5 [5, 13, 20]. Therefore, measurements were grouped according to age cohorts (2 to 11 years) and time after KT using yearly intervals. This allowed us to create two sets of correlated outcomes for statistical analysis. A total of 722 annual measurements (mean 4.9 per child) were available.

Linear mixed-effects models were used to assess predictors (defined as factors and covariates) of growth outcome and calculate adjusted and non-adjusted mean *Z* scores for linear body dimensions and proportions. *Factors:* congenital CKD (yes versus no), pre-emptive KT versus prior dialysis, living versus deceased donor, SGA history (yes versus no), sex (female versus male), and prior GH treatment (yes versus no). Due to the low frequency of GH treatment after KT (4%) this parameter was not included in the statistical analysis. *Covariates*: age at CKD 5, age at KT, time after KT, birth weight for length, umbilical cord artery pH at birth, parental height, as well as steroid dosage, eGFR, bicarbonate, and hemoglobin levels after KT.

**Fig. 1:**
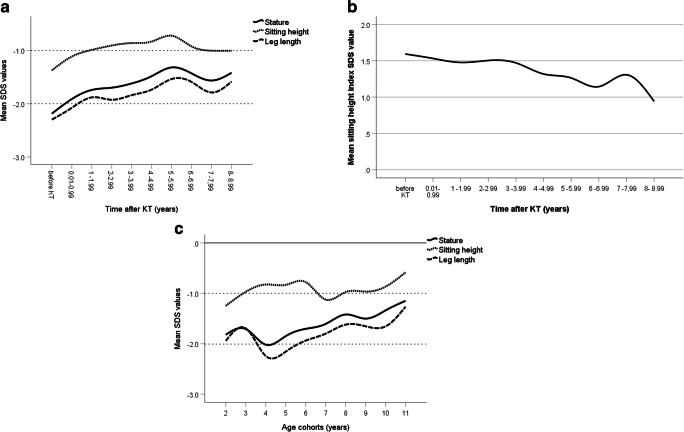
Post-transplant growth in 148 prepubertal children by the time after kidney transplantation (**a**, **b**), and by age cohorts (**c**). **a** mean *Z* scores for height, sitting height, and leg length. All linear body dimensions were significantly reduced at baseline compared to healthy children (each *p* < 0.001), the degree of impairment significantly differed between the three body dimensions, indicating disproportionate stunting (each *p* < 0.001); all body dimensions increased after kidney transplantation (KT), which became significant after the 1st year (sitting height) and 3rd year (leg length), respectively (each *p* < 0.05 versus baseline). The lower dotted horizontal line refers to the lower normal range (− 2.0 SD score). **b** mean *Z* scores for sitting height index (ratio between sitting height and total body height) as a measure for body disproportion. Mean sitting height index was significantly increased before KT compared to healthy children and continuously decreased after KT which reached the level of statistical significance from the 6th year onwards. **c** mean *Z* scores for height, sitting height, and leg length height by age cohorts. Catch-up growth after KT was mainly due to increased standardized sitting height in young children (< 4 years), and improved standardized leg length in older children. The lower dotted horizontal line refers to the lower normal range (− 2.0 SD score)

**Fig. 2:**
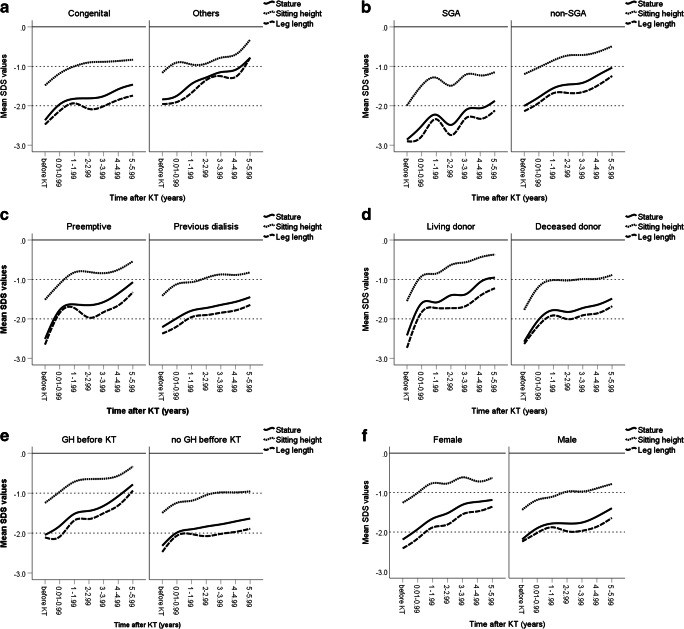
Mean *Z* scores SD for height, sitting height, and leg length before and after kidney transplantation (KT) by **a** primary disease (congenital CKD (*n* = 117) versus others (*n* = 31)); **b** small for gestational status (SGA, *n* = 37) versus others (*n* = 98), type of KT; **c** pre-emptive KT (*n* = 52) versus prior dialysis (*n* = 96); **d** living (*n* = 43) versus deceased KT (*n* = 103); **e** use of growth hormone treatment prior to KT (yes (*n* = 50) versus no (*n* = 92); and **f** male (*n* = 98) versus female (*n* = 50). SDS, SD score; GH, growth hormone. Data were not adjusted for confounders

The SPSS MMX-repeated measurement procedure defined as Kronecker product covariance structures was used, where covariance matrices are adjusted to respective time scales: one time scale with origin defined by time of birth and the other scale with origin at the age at KT. Finding the best Kronecker product structure for our data required selecting models for each of the time repeated factors. An unstructured covariance matrix for age cohort paired with also unstructured time after KT was chosen. A cubic spline function was used in Figs. 1 and 2 for graphical presentation of linear growth. The standard statistical package SPSS for Windows, version 26.0 (IBM Corporation, New York, USA) was used for statistical calculations. Results were considered significant at a level of *p* < 0.05.

## Results

Key characteristics of the study cohort are given in Table 1. Median age at CKD 5 and KT was 2.8 years and 3.7 years, respectively. In agreement with the young age at the time of CKD 5, the majority of patients suffered from congenital CKD (79.1%) mostly related to congenital anomalies of the kidneys and urinary tract (48%), which probably explains the high proportion of boys (62.2%). Twenty-seven percent of patients were born SGA, and 35.1% and 29.3% of patients underwent pre-emptive and/or living donor KT, respectively. Growth hormone was commenced in the pre-transplant period in 33.8% of patients over a median period of 1.4 years (IQR 0.57–2.23), and discontinued at the time of KT in all patients. Mean eGFR and steroid-dosage was 59 ml/min per 1.73 m^2^ and 0.1 mg/kg per day, respectively. Metabolic acidosis (bicarbonate < 22 mmol/L) and mild (hemoglobin < 12 g/dl) or moderate (hemoglobin < 10 g/dl) anemia was noted in 35.1%, 72.1%, and 14.9% of annual measurements, respectively.

### Body dimensions and proportions before kidney transplantation

Pre-transplant mean *Z* scores for all linear body dimensions were reduced: height − 2.18 ± 1.08, sitting height − 1.37 ± 0.97, leg length − 2.30 ± 1.17 (each *p* < 0.001 versus healthy children, Fig. 1a). Higher impairment of leg length compared to sitting height (*p* < 0.001) resulted in a markedly elevated sitting height index (1.59 ± 1.1 SDS, *p* < 0.001 versus healthy children, Fig. 1b), indicating disproportionate short stature.

### Changes of body dimensions and proportions after transplantation

The increase in standardized height was most pronounced during the early post-transplant years, and amounted to 0.53 SDS and 0.84 SDS at 2 and 5 years, respectively (each *p* < 0.01 versus baseline Fig. 1a). Accordingly, the percentage of patients with short stature (height ≤ − 2.0 SDS) decreased from 61.8% at baseline to 38.0% and 28.2% at 2 and 5 years, respectively (each *p* < 0.01). The degree and timing of catch-up growth of linear body segments, i.e., trunk and legs, differed distinctly. Although both standardized leg and trunk length significantly increased during the first 2 years after KT (*p* < 0.01), the increase in sitting height clearly exceeded that of leg length (0.68 SDS versus 0.41 SDS, *p* < 0.05). Thereafter, no significant changes in sitting height SDS were noted, whereas standardized leg length continuously further increased resulting in sustained improvement of sitting height index (Fig. 1b). The latter reached the level of statistical significance from the 6th year onwards when compared to baseline values (Fig. 1b). Accordingly, the percentage of patients with abnormal body proportion, defined as sitting height index ≥ 2 SDS, decreased from 35.8% at baseline to 20.6% at 5 years (*p* < 0.05).

Age-related changes in body dimensions after KT are given in Fig. 1c. Two distinct age periods were noted, i.e., before and after 4 years of age, reflecting the transition from toddlerhood to preschool age. Sitting height SDS increased continuously before the age of 4 years, while leg length SDS decreased (each *p* < 0.05) which explains the lack of catch-up growth in terms of body height and indicates progressive body disproportion. By contrast, a sustained increase in both standardized leg length and body height was noted after the age of 4 years (each *p* < 0.05, Fig. 1c) while sitting height SDS even decreased from 6 to 7 years of age, and remained constant thereafter indicating improvement of body proportions (each *p* < 0.05 for age cohort 5 and above versus age 4 years).

### Determinants of post-transplant growth

Figure 2 illustrates non-adjusted post-transplant growth in specific subgroups. Impairment of linear body dimensions before KT was higher in children with SGA history compared to those with no SGA history (each *p* < 0.05), but did not significantly differ in other subgroups. Linear body dimensions after KT significantly differed in all presented subgroups (each *p* < 0.05) except for sitting height after pre-emptive KT compared to prior dialysis and for stature and leg length in male compared to female patients (Table 2).

**Table 2:** Non-adjusted anthropometric parameters in subgroups of a cohort of 148 prepubertal kidney transplant recipients

Parameter	Height SDS	*p* value	Leg length SDS	*p* value	Sitting height SDS	*p* value	Sitting height index SDS	*p* value
Congenital CKD Others	− 1.65 (− 1.76 to − 1.54) − 1.11 (− 1.33 to − 0.88)	0.000	− 1.85 (− 1.97 to − 1.73) − 1.24 (− 1.48 to − 1.00)	0.000	− 0.88 (− 0.98 to − 0.78) − 0.65 (− 0.86 to − 0.45)	0.031	1.50 (1.38 to 1.61) 0.88 (0.65 to 1.11)	0.000
SGA non-SGA	− 1.73 (− 1.92 to − 1.54) − 1.04 (− 1.18 to − 0.91)	0.000	− 1.87 (− 2.08 to − 1.67) − 1.21 (− 1.36 to − 1.07)	0.000	− 1.04 (− 1.21 to − 0.86) − 0.49 (− 0.62 to − 0.37)	0.000	1.35 (1.15 to 1.55) 1.03 (0.89 to 1.17)	0.002
Pre-emptive KT Previous dialysis	− 1.28 (− 1.47 to − 1.09) − 1.49 (− 1.62 to − 1.35)	0.028	− 1.44 (− 1.65 to − 1.24) − 1.65 (− 1.79 to − 1.50)	0.048	− 0.69 (− 1.86 to − 0.52) − 0.84 (− 1.97 to − 0.72)	0.08	1.11 (0.91 to 1.31) 1.27 (1.13 to 1.41)	0.105
GH before KT No GH before KT	− 1.15 (− 1.32 to − 0.98) − 1.62 (− 1.77 to − 1.47)	0.000	− 1.30 (− 1.48 to − 1.12) − 1.79 (− 1.95 to − 1.63)	0.000	− 0.55 (− 0.70 to − 0.39) − 0.98 (− 1.12 to − 0.84)	0.000	1.11 (0.93 to 1.29) 1.27 (1.11 to 1.42)	0.090
Living donor KT Deceased donor KT	− 1.16 (− 1.35 to − 0.97) − 1.61 (− 1.74 to − 1.47)	0.000	− 1.35 (− 1.56 to − 1.15) − 1.74 (− 1.88 to − 1.59)	0.000	− 0.59 (− 0.77 to − 0.42) − 0.94 (− 1.06 to − 0.82)	0.000	1.09 (0.88 to 1.29) 1.29 (1.15 to 1.43)	0.038
Female Male	− 1.33 (− 1.51 to − 1.17) − 1.43 (− 1.59 to − 1.28)	0.290	− 1.52 (− 1.70 to − 1.33) − 1.57 (− 1.74 to − 1.41)	0.575	− 0.68 (− 0.83 to − 0.52) − 0.86 (− 1.00 to − 0.71)	0.033	1.24 (1.06 to 1.41) 1.14 (0.98 to 1.31)	0.346

Data are presented as SD score (SDS) values, estimated marginal means (95% confidence intervals). *p* values are based on the linearly independent pairwise comparisons among the estimated marginal means

In the multivariate analysis, congenital CKD was significantly associated with sitting height index (Table 3). Time after KT was associated with height, leg length, and sitting height index. The use of GH in the pre-transplant period, steroid dosage, eGFR, SGA history, and parental height were associated with all linear body dimensions after KT (each *p* < 0.05), whereas birth weight for length and umbilical cord artery pH were associated with body height and sitting height only. By contrast, the type of KT, age at CKD 5 or KT, and hemoglobin and plasma HCO_3_ levels were not associated with growth outcome.

**Table 3:** Linear mixed-effects models of determinants of adjusted *Z* scores for anthropometric parameters in 148 kidney transplant recipients

Parameter	Height	Leg length	Sitting height	Sitting height index
Congenital CKD^a^	− 0.42 (− 0.93 to 0.08)	− 0.50 (− 1.07 to − 0.07)	− 0.15 (− 0.58 to 0.29)	0.68^i^ (0.10 to 1.27)
Pre-emptive KT^b^	− 0.19 ( − 0.70 to 0.33)	0.11 (−0.69 to 0.47)	− 0.26 (− 0.70 to 0.19)	− 0.14 (− 0.74 to 0.46)
Living donor KT^c^	0.16 (0.25 to 0.57)	0.03 (− 0.43 to 0.49)	0.14 (− 0.22 to 0.49)	− 0.09 (− 0.57 to 0.38)
Gender^d^	0.02 (− 0.34 to 0.38)	0.01 (− 0.40 to 0.42)	0.05 (− 0.26 to 0.36)	0.12 (− 0.35 to 0.52)
Age at CKD 5 (in year)	0.18 (− 0.05 to 0.40)	0.20 (− 0.05 to 0.45)	0.16 (− 0.03 to 0.35)	− 0.09 (− 0.35 to 0.17)
Age at KT (in year)	− 0.11 (− 0.35 to 0.14)	− 0.15 (− 0.42 to 0.13)	− 0.17 (− 0.38 to 0.04)	0.03 (− 0.26 to 0.32)
Total time after KT (in 10 * year)	0.11^g^ (0.08 to 0.13)	1.04^g^ (0.78 to 1.30)	0.18 (− 0.06 to 0.43)	− 0.69^g^ (− 0.97 to 0.40)
GH before KT^e^	0.47 (0.09 to 0.85)	0.49 (0.06 to 0.92)	0.37 (0.04 to 0.70)	− 0.34 (− 0.79 to 0.10)
Steroid dosage (in mg/kg)	− 0.83^g^ (− 1.45 to − 0.20)	− 1.11^g^ (− 1.82 to − 0.40)	− 0.65^h^ (− 1.30 to − 0.01)	0.88 (− 0.03 to 1.79)
Hemoglobin (in 10 * g/dl)	0.32 (0.10 to 0.75)	0.28 (− 0.20 to 0.75)	− 0.25 (− 0.20 to 0.70)	− 0.38 (− 0.97 to 0.22)
Plasma HCO_3_ (in 10 * mmol/l)	0.12 (0.16 to 0.40)	0.08 (− 0.22 to 0.38)	− 0.10 (− 0.19 to 0.39)	− 0.14 (− 0.24 to 0.52)
eGFR (in 100 * ml/min per 1.73 m^2^)	0.65^g^ (0.45 to − 0.85)	0.57^g^ (0.35 to 0.80)	0.63^g^ (− 0.41 to 0.85)	0.01 (− 0.28 to 0.30)
Small for gestational age history^f^	− 0.74^g^ (− 1.19 to − 0.30)	− 0.74^g^ (− 1.25 to − 0.24)	− 0.60^g^ (− 0.98 to −0.22)	0.25 (− 0.27 to 0.77)
Birth weight for length (in 100 * g/cm)	1.95^h^ (0.35 to 3.55)	1.34 (0.48 to 3.15)	1.86^g^ (0.47 to 3.24)	− 0.60 (− 2.48 to 1.28)
Umbilical cord artery pH	2.43^h^ (0.02 to 4.83)	1.69 (− 1.04 to 4.43)	2.54^h^ (0.44 to 4.63)	0.07 (− 2.74 to 2.89)
Parental height (in 1000 * cm)	0.17^g^ (0.09 to 0.26)	0.18^g^ (0.08 to 0.28)	0.15^g^ (0.08 to 0.23)	− 0.06 (− 0.17 to 0.04)

Data are presented as *ß* values (95% confidence intervals). ^a^Congenital CKD (1) versus others (0); The numbers in parentheses indicate the dummy variable used for parameters of the LMM model. ^b^Pre-emptive KT (0) versus previous dialysis treatment (1); ^c^Living-related donor graft (LRD = 0) versus deceased donor graft (DD = 1); ^d^Gender (female = 0, male = 1); ^e^Growth hormone treatment before KT (0) versus others (1); ^f^Small for gestational age history (0) versus others (1). ^g^*p* < 0.01; ^h^*p* < 0.05

Next, we adjusted anthropometric data for age at CKD 5 and at KT, time after KT, steroid dosage, hemoglobin and HCO_3_ levels, eGFR, birth weight for length, umbilical cord pH, and parental height, to estimate the specific impact of certain dichotomous clinical parameters on post-transplant growth (Table 4). All linear body dimensions were significantly impaired in patients with a history of SGA but were superior in patients with prior GH treatment when compared to their respective controls (each *p* < 0.05). Patients with congenital CKD showed a higher degree of body disproportion compared to their peers (*p* < 0.05). By contrast, neither the mode of transplantation nor sex turned out to be significant determinants of post-transplant growth after adjustment for confounders.

**Table 4:** Adjusted anthropometric parameters in subgroups of a cohort of 148 prepubertal kidney transplant recipients

Parameter	Height SDS	*p* value	Leg length SDS	*p* value	Sitting height SDS	*p* value	Sitting height index SDS	*p* value
Congenital CKD Others	− 1.79 (− 2.05 to − 1.53) − 1.37 (− 1.88 to − 0.86)	0.097	− 2.02 (− 2.31 to − 1.73) − 1.52 (− 2.10 to − 0.94)	0.083	− 0.98 (− 1.21 to − 0.76) − 0.83 (− 1.28 to − 0.39)	0.500	1.59 (1.28 to 1.89) 0.90 (0.30 to 1.50)	0.023
SGA non-SGA	− 1.95 (− 2.41 to − 1.49) − 1.21 (− 1.50 to − 0.92)	0.001	− 2.14 (− 2.67 to − 1.61) − 1.40 (− 1.73 to − 1.06)	0.004	− 1.21 (− 1.61 to − 0.81) − 0.61 (− 0.86 to − 0.35)	0.002	1.37 (0.83 to 1.91) 1.12 (0.77 to 1.46)	0.342
Pre-emptive KT Previous dialysis	− 1.67 (− 2.16 to − 1.19) − 1.49 (− 1.80 to − 1.18)	0.475	− 1.82 (− 2.38 to − 1.27) − 1.71 (− 2.07 to − 1.36)	0.710	− 1.04 (− 1.46 to − 0.62) − 0.78 (− 1.05 to − 0.51)	0.255	1.17 (0.60 to 1.74) 1.31 (0.95 to 1.53)	0.639
GH before KT No GH before KT	− 1.35 (− 1.73 to − 0.96) − 1.82 (− 2.17 to − 1.46)	0.016	− 1.52 (− 1.97 to − 1.08) − 2.01 (− 2.42 to − 1.61)	0.026	− 0.73 (− 1.06 to − 0.39) − 1.09 (− 1.40 to − 0.79)	0.030	1.07 (0.62 to 1.90) 1.41 (1.00 to 1.83)	0.131
Living donor KT Deceased donor KT	− 1.50 (− 1.95 to − 1.06) − 1.66 (− 1.96 to − 1.36)	0.442	− 1.75 (− 2.26 to − 1.25) − 1.78 (− 2.12 to − 1.45)	0.896	− 0.84 (− 1.22 to − 0.45) − 0.98 (− 1.23 to − 0.72)	0.436	1.20 (0.67 to 1.72) 1.29 (0.94 to 1.63)	0.695
Female Male	− 1.57 (− 1.95 to − 1.19) − 1.59 (− 1.94 to − 1.24)	0.921	− 1.77 (− 2.20 to − 1.34) − 1.77 (− 2.17 to − 1.37)	0.979	− 0.88 (− 1.21 to − 0.56) − 0.93 (− 1.24 to − 0.63)	0.763	1.30 (0.86 to 1.75) 1.18 (0.77 to 1.59)	0.570

Data are presented as estimated marginal means (95% confidence intervals); *SDS*, SD score; *SGA*, small for gestational age; *KT*, kidney transplantation; *GH*, growth hormone. Data were adjusted for age at stage 5 chronic kidney disease, age at KT, steroid dosage, time after KT, hemoglobin and HCO_3_ levels, estimated glomerular filtration rate, birth weight for length, umbilical cord pH, and parental height. *p* values are based on the linearly independent pairwise comparisons among the estimated marginal means

## Discussion

The main findings of our study suggest that catch-up growth after KT in young children (< 4 years) is mainly due to stimulated trunk growth and improved leg growth in older children, resulting in normalization of body height and proportions before puberty in the vast majority of patients. In addition to transplant function and steroid exposure, use of GH in the pre-transplant period, congenital CKD, birth parameters, and parental height were significantly associated with growth outcome. Although, unadjusted growth data suggested superior post-transplant growth after (pre-emptive) living donor KT, this was no longer detectable after adjusting for the abovementioned confounders.

As expected, our young patient cohort (age at KT < 8 years) presented with marked growth retardation at the time of KT with a mean standardized height of − 2.18 SDS, which was lower compared to that in recent reports from North American (− 1.73 SDS) and European (− 1.77 SDS) registries most likely due to the high percentage of children with congenital CKD (79%) [5, 9]. Consequently, and in line with our previous studies, 38% of patients presented with abnormal body proportions due to a more severe impairment of leg compared to trunk length, supporting the concept that CKD 5 in young age results in disproportionate short stature; this is similarly observed in other genetic diseases affecting the skeleton, e.g., skeletal dysplasia, X-linked hypophosphatemia, and Ullrich Turner syndrome or chronic illness [25, 28–31].

Catch-up growth after KT was highest during the early post-transplant years, and amounted to 0.53 SDS and 0.84 SDS at 2 and 5 years, which was slightly higher than those reported from European and North American registries ranging from 0.5 to 0.7 SDS within 5 years after KT in prepubertal children. This may be at least partly related to their higher initial growth deficit in our patient cohort [32]. Consequently, the percentage of patients with normal height (≥ 2 SD) increased from 38.2% at the time of KT to 78.3% at the age of 11 years. Nevertheless, we have to state that catch-up growth until pubertal age was incomplete in 21.7% of patients. Since pubertal growth is usually impaired in pediatric KT patients, due to a delayed and shortened growth spurt, this illustrates the importance of growth-promoting measures prior to KT such as adequate nutrition, correction of metabolic acidosis, and GH treatment [1, 33, 34].

Interestingly, the degree of catch-up growth of linear body segments, i.e., trunk and legs, was clearly age dependent. In children aged less than 4 years, trunk catch-up growth was more pronounced compared to leg catch-up growth, which was significant later on only. Thus, catch-up growth in young children primarily concerned a normalization of trunk length, while leg length and thus body proportions were only improved in later years. These data might reflect an evolutionary principle of preferred growth of body segments containing vital organs (the trunk) in periods of catch-up growth. The sequence of trunk and leg catch-up growth in the present study resembles the situation in healthy children where trunk growth precedes leg growth in early childhood resulting in both normalization of height and body proportions in the majority of patients before pubertal age [26]. The latter finding is of importance as our previous analysis of growth after KT in a mixed cohort of prepubertal and pubertal children suggested that improvements in body proportions are only to be expected during pubertal age [13]. A reversible within-variation of organ systems or body segments as seen in the present study is called “phenotypic flexibility/plasticity” and seems to be of evolutionary benefit by allowing an individual to survive in case of deterioration of living conditions and enabling increased chances of reproduction after improved environmental conditions or illness, e.g., as seen here in children with CKD 5 undergoing KT [35, 36].

In the multivariable analysis, time after KT, allograft function, corticosteroid dosage, congenital CKD, birth parameters, and parental height were significantly associated with post-transplant growth, which is in line with previous studies [5, 9, 11–14, 37]. The average prednisolone dosage during the study period amounted to 3 mg/m^2^ per day and steroid withdrawal was done in 18% of patients, only. Thus, a more vigorous steroid-withdrawal or use of steroid-free free immunosuppressive protocols might have resulted in superior growth outcome as suggested by recent trials [11, 12, 14, 37].

Analysis of unadjusted data revealed superior growth outcome after (pre-emptive) living donor KT with respect to all linear body dimensions. However, this did not hold true after adjusting for confounders including age at CKD 5, age at KT, time after KT, steroid dosage, eGFR, plasma HCO_3_, hemoglobin, birth parameters and parental height. Our data suggest that the superior growth outcome in children undergoing pre-emptive or living donor KT reported from some but not all uncontrolled studies is probably not related to the procedure per se but rather due to associated factors like better graft function, lower steroid exposure, and/or better-preserved growth at the time of KT in these patients [5, 6, 9, 15]. Nevertheless, (pre-emptive) living donor KT remains the treatment of choice in children with CKD 5 due to its superior outcome with respect to neurocognitive development, quality of life, and cardiovascular health [38–40].

It is our policy to apply GH treatment in all children with CKD stages 3–5D presenting with persistent growth failure based on current guidelines [1, 41]. Consequently 34% of our patients attaining CKD 5 at young age (2.8 years) were started on GH prior to KT, but stopped in all patients at the time of transplantation. It was hypothesized that this approach may impact on post-transplant growth [1]. Here we could clearly demonstrate that GH treatment in the pre-transplant period is significantly associated with superior growth outcomes after KT (increase in height, 0.47 SDS; leg length, 0.49; sitting height, 0.36 SDS) even after adjustment for confounders, supporting the vigorous use of GH in short children with CKD [1, 41–43].

Our study has several limitations and strengths. First, the number of patients in some subgroups was rather small. Second, due to the low frequency of GH use in the post-transplant period (4%), this parameter could not be incorporated in the multivariate analysis. Third, socioeconomic status which has been shown to be associated with treatment adherence and patient outcome after KT could not be addressed in the present study [44]. Fourth, several other parameters potentially affecting growth before and/or after KT, such as age at onset of CKD and years during significant growth impacting CKD, parathyroid hormone, albumin and sodium levels, and data on nutritional adequacy could not be addressed in our study. However, the prospective comprehensive anthropometric assessment, collection of major biochemical and clinical parameters, exclusion of pubertal patients, and long follow-up enabled to us analyze the age-dependent impact of KT on body dimensions and proportion and its determinants independent of main confounders.

In conclusion, catch-up growth after KT is mainly due to stimulated trunk growth in young children (< 4 years), and improved leg growth in older children, resulting in normalization of body height and proportions until pubertal age in the vast majority of patients. Beside transplant function, steroid exposure and use of GH in the pre-transplant period are the main potentially modifiable factors associated with better growth after KT. Therefore, clinical management should focus on these factors. In addition, given risk factors for poor growth outcome like abnormal birth history, congenital CKD, and low parental height should be taken in account when rendering a decision whether to initiate such growth-promoting measures in order to maximize growth outcomes.

## References

[1] Drube J, Wan M, Bonthuis M, Wühl E, Bacchetta J, Santos F, Grenda R, Edefonti A, Harambat J, Shroff R, Tönshoff B, Haffner D, European Society for Paediatric Nephrology Chronic Kidney Disease Mineral and Bone Disorders, Dialysis, and Transplantation Working Groups (2019). Clinical practice recommendations for growth hormone treatment in children with chronic kidney disease. Nat Rev Nephrol.

[2] Franke D, Winkel S, Gellermann J, Querfeld U, Pape L, Ehrich JH, Haffner D, Pavicic L, Zivicnjak M (2013). Growth and maturation improvement in children on renal replacement therapy over the past 20 years. Pediatr Nephrol.

[3] Harambat J, Bonthuis M, van Stralen KJ, Ariceta G, Battelino N, Bjerre A, Jahnukainen T, Leroy V, Reusz G, Sandes AR, Sinha MD, Groothoff JW, Combe C, Jager KJ, Verrina E, Schaefer F, for the ESPN/ERA-EDTA Registry (2013). Adult height in patients with advanced CKD requiring renal replacement therapy during childhood. Clin J Am Soc Nephrol.

[4] Al-Uzri A, Matheson M, Gipson DS, Mendley SR, Hooper SR, Yadin O, Rozansky DJ, Moxey-Mims M, Furth SL, Warady BA, Gerson AC, Chronic Kidney Disease in Children Study Group (2013). The impact of short stature on health-related quality of life in children with chronic kidney disease. J Pediatr.

[5] Fine RN, Martz K, Stablein D (2010). What have 20 years of data from the north american pediatric renal transplant cooperative study taught us about growth following renal transplantation in infants, children, and adolescents with end-stage renal disease?. Pediatr Nephrol.

[6] Chua A, Cramer C, Moudgil A, Martz K, Smith J, Blydt-Hansen T, Neu A, Dharnidharka VR, NAPRTCS investigators (2019). Kidney transplant practice patterns and outcome benchmarks over 30 years: the 2018 report of the NAPRTCS. Pediatr Transplant.

[7] Ku E, Fine RN, Hsu CY, McCulloch C, Glidden DV, Grimes B, Johansen KL (2016). Height at first RRT and mortality in children. Clin J Am Soc Nephrol.

[8] Wong CS, Gipson DS, Gillen DL, Emerson S, Koepsell T, Sherrard DJ, Watkins SL, Stehman-Breen C (2000). Anthropometric measures and risk of death in children with end-stage renal disease. Am J Kidney Dis.

[9] Bonthuis M, Groothoff JW, Ariceta G, Baiko S, Battelino N, Bjerre A, Cransberg K, Kolvek G, Maxwell H, Miteva P, Molchanova MS, Neuhaus TJ, Pape L, Reusz G, Rousset-Rouviere C, Sandes AR, Topaloglu R, Van Dyck M, Ylinen E, Zagozdzon I, Jager KJ, Harambat J (2020). Growth patterns after kidney transplantation in european children over the past 25 years: an ESPN/ERA-EDTA registry study. Transplantation.

[10] Winterberg PD, Garro R (2019). Long-term outcomes of kidney transplantation in children. Pediatr Clin N Am.

[11] Webb NJ, Douglas SE, Rajai A, Roberts SA, Grenda R, Marks SD, Watson AR, Fitzpatrick M, Vondrak K, Maxwell H, Jaray J, Van Damme-Lombaerts R, Milford DV, Godefroid N, Cochat P, Ognjanovic M, Murer L, McCulloch M, Tonshoff B (2015). Corticosteroid-free kidney transplantation improves growth: 2-year follow-up of the TWIST randomized controlled trial. Transplantation.

[12] Tsampalieros A, Knoll GA, Molnar AO, Fergusson N, Fergusson DA (2017). Corticosteroid use and growth after pediatric solid organ transplantation: a systematic review and meta-analysis. Transplantation.

[13] Franke D, Thomas L, Steffens R, Pavičić L, Gellermann J, Froede K, Querfeld U, Haffner D, Živičnjak M (2015). Patterns of growth after kidney transplantation among children with ESRD. Clin J Am Soc Nephrol.

[14] Sarwal MM, Ettenger RB, Dharnidharka V, Benfield M, Mathias R, Portale A, McDonald R, Harmon W, Kershaw D, Vehaskari VM, Kamil E, Baluarte HJ, Warady B, Tang L, Liu J, Li L, Naesens M, Sigdel T, Waskerwitz J, Salvatierra O (2012). Complete steroid avoidance is effective and safe in children with renal transplants: a multicenter randomized trial with three-year follow-up. Am J Transplant.

[15] Pape L, Ehrich JH, Zivicnjak M, Offner G (2005). Growth in children after kidney transplantation with living related donor graft or cadaveric graft. Lancet.

[16] Greenbaum LA, Munoz A, Schneider MF, Kaskel FJ, Askenazi DJ, Jenkins R, Hotchkiss H, Moxey-Mims M, Furth SL, Warady BA (2011). The association between abnormal birth history and growth in children with CKD. Clin J Am Soc Nephrol.

[17] Franke D, Alakan H, Pavicic L, Gellermann J, Muller D, Querfeld U, Haffner D, Zivicnjak M (2013). Birth parameters and parental height predict growth outcome in children with chronic kidney disease. Pediatr Nephrol.

[18] Franke D, Volker S, Haase S, Pavicic L, Querfeld U, Ehrich JH, Zivicnjak M (2010). Prematurity, small for gestational age and perinatal parameters in children with congenital, hereditary and acquired chronic kidney disease. Nephrol Dial Transplant.

[19] Franke D, Steffens R, Thomas L, Pavicic L, Ahlenstiel T, Pape L, Gellermann J, Muller D, Querfeld U, Haffner D, Zivicnjak M (2017). Kidney transplantation fails to provide adequate growth in children with chronic kidney disease born small for gestational age. Pediatr Nephrol.

[20] Zivicnjak M, Franke D, Filler G, Haffner D, Froede K, Nissel R, Haase S, Offner G, Ehrich JH, Querfeld U (2007). Growth impairment shows an age-dependent pattern in boys with chronic kidney disease. Pediatr Nephrol.

[21] De Souza VC, Rabilloud M, Cochat P, Selistre L, Hadj-Aissa A, Kassai B, Ranchin B, Berg U, Herthelius M, Dubourg L (2012). Schwartz formula: is one k-coefficient adequate for all children?. PLoS One.

[22] Voigt M, Schneider KT, Jährig K (1996). Analysis of a 1992 birth sample in Germany. 1: new percentile values of the body weight of newborn infants. Geburtshilfe Frauenheilkd.

[23] Tanner JM, Goldstein H, Whitehouse RH (1970). Standards for children’s height at age 2 to 9 years allowing for height of parents. Arch Dis Child.

[24] Weiner J, Lourie J (1981). Practical human biology.

[25] Zivicnjak M, Schnabel D, Staude H, Even G, Marx M, Beetz R, Holder M, Billing H, Fischer DC, Rabl W, Schumacher M, Hiort O, Haffner D, Hypophosphatemic Rickets Study Group of the Arbeitsgemeinschaft fur Padiatrische Endokrinologie and Gesellschaft fur Padiatrische Nephrologie (2011). Three-year growth hormone treatment in short children with X-linked hypophosphatemic rickets: effects on linear growth and body disproportion. J Clin Endocrinol Metab.

[26] Zivicnjak M, Narancic NS, Szirovicza L, Franke D, Hrenovic J, Bisof V (2003). Gender-specific growth patterns for stature, sitting height and limbs length in croatian children and youth (3 to 18 years of age). Coll Antropol.

[27] Zivicnjak M, Smolej Narancić N, Szirovicza L, Franke D, Hrenović J, Bisof V, Tomas Z, Skarić-Jurić T (2008). Gender-specific growth patterns of transversal body dimensions in croatian children and youth (2 to 18 years of age). Coll Antropol.

[28] Zivicnjak M, Franke D, Zenker M, Hoyer J, Lucke T, Pape L, Ehrich JH (2009). *SMARCAL1* mutations: a cause of prepubertal idiopathic steroid-resistant nephrotic syndrome. Pediatr Res.

[29] Delvecchio M, Cavallo L (2010). Growth and endocrine function in thalassemia major in childhood and adolescence. J Endocrinol Investig.

[30] Mazzanti L, Matteucci C, Scarano E, Tamburrino F, Ragni MC, Cicognani A (2010). Auxological and anthropometric evaluation in skeletal dysplasias. J Endocrinol Investig.

[31] Malaquias AC, Scalco RC, Fontenele EG, Costalonga EF, Baldin AD, Braz AF, Funari MF, Nishi MY, Guerra-Junior G, Mendonca BB, Arnhold IJ, Jorge AA (2013). The sitting height/height ratio for age in healthy and short individuals and its potential role in selecting short children for SHOX analysis. Horm Res Paediatr.

[32] Nissel R, Brazda I, Feneberg R, Wigger M, Greiner C, Querfeld U, Haffner D (2004). Effect of renal transplantation in childhood on longitudinal growth and adult height. Kidney Int.

[33] Shaw V, Polderman N, Renken-Terhaerdt J, Paglialonga F, Oosterveld M, Tuokkola J, Anderson C, Desloovere A, Greenbaum L, Haffner D, Nelms C, Qizalbash L, Vande Walle J, Warady B, Shroff R, Rees L (2020). Energy and protein requirements for children with CKD stages 2-5 and on dialysis-clinical practice recommendations from the pediatric renal nutrition taskforce. Pediatr Nephrol.

[34] Behnisch R, Kirchner M, Anarat A, Bacchetta J, Shroff R, Bilginer Y, Mir S, Caliskan S, Paripovic D, Harambat J, Mencarelli F, Büscher R, Arbeiter K, Soylemezoglu O, Zaloszyc A, Zurowska A, Melk A, Querfeld U, Schaefer F, 4C Study Consortium (2019) Determinants of statural growth in European children with chronic kidney disease: findings from the cardiovascular comorbidity in children with chronic kidney disease (4C) study. Front Pediatr 7:278 10.3389/fped.2019.00278 PMC6625460 31334210

[35] Price TD, Qvarnstrom A, Irwin DE (2003). The role of phenotypic plasticity in driving genetic evolution. Proc Biol Sci.

[36] Piersma T, Drent J (2003). Phenotypic felxibility and the evolution of orgasnismal design. Trends Ecol Evol.

[37] Grenda R, Watson A, Trompeter R, Tonshoff B, Jaray J, Fitzpatrick M, Murer L, Vondrak K, Maxwell H, van Damme-Lombaerts R, Loirat C, Mor E, Cochat P, Milford DV, Brown M, Webb NJ (2010). A randomized trial to assess the impact of early steroid withdrawal on growth in pediatric renal transplantation: the TWIST study. Am J Transplant.

[38] Hartmann H, Hawellek N, Wedekin M, Vogel C, Das AM, Balonwu K, Ehrich JH, Haffner D, Pape L (2015). Early kidney transplantation improves neurocognitive outcome in patients with severe congenital chronic kidney disease. Transpl Int.

[39] Bertram JF, Goldstein SL, Pape L, Schaefer F, Shroff RC, Warady BA (2016). Kidney disease in children: latest advances and remaining challenges. Nat Rev Nephrol.

[40] Schmidt BMW, Sugianto RI, Thurn D, Azukaitis K, Bayazit AK, Canpolat N, Eroglu AG, Caliskan S, Doyon A, Duzova A, Karagoz T, Anarat A, Deveci M, Mir S, Ranchin B, Shroff R, Baskin E, Litwin M, Ozcakar ZB, Buscher R, Soylemezoglu O, Dusek J, Kemper MJ, Matteucci MC, Habbig S, Laube G, Wuhl E, Querfeld U, Sander A, Schaefer F, Melk A, 4C Study Consortium (2018). Early effects of renal replacement therapy on cardiovascular comorbidity in children with end-stage kidney disease: Findings from the 4C-T study. Transplantation.

[41] Mahan JD, Warady BA, Consensus Committee (2006). Assessment and treatment of short stature in pediatric patients with chronic kidney disease: a consensus statement. Pediatr Nephrol.

[42] Akchurin OM, Kogon AJ, Kumar J, Sethna CB, Hammad HT, Christos PJ, Mahan JD, Greenbaum LA, Woroniecki R (2017). Approach to growth hormone therapy in children with chronic kidney disease varies across North America: the Midwest pediatric nephrology consortium report. BMC Nephrol.

[43] Akchurin OM, Schneider MF, Mulqueen L, Brooks ER, Langman CB, Greenbaum LA, Furth SL, Moxey-Mims M, Warady BA, Kaskel FJ, Skversky AL (2014). Medication adherence and growth in children with CKD. Clin J Am Soc Nephrol.

[44] Francis A, Didsbury M, Lim WH, Kim S, White S, Craig JC, Wong G (2016). The impact of socioeconomic status and geographic remoteness on access to pre-emptive kidney transplantation and transplant outcomes among children. Pediatr Nephrol.

